# Assessing the welfare of smallholder pigs in Vietnam using a pilot protocol

**DOI:** 10.1007/s11250-025-04602-5

**Published:** 2025-09-23

**Authors:** Sinh Dang-Xuan, Le Thi Thanh Huyen, Tu-Quynh Ha, Do Van Duc, Le Tien Dung, Han Anh Tuan, Fred Unger, Rebecca Doyle, Jenny-Ann Toribio

**Affiliations:** 1 International Livestock Research Institute, Hanoi, Vietnam; 2 National Institute of Animal Science, Hanoi, Vietnam; 3 The Royal (Dick) School of Veterinary Studies, The University of Edinburgh, Edinburgh, Scotland; 4 International Livestock Research Institute, Addis Ababa, Ethiopia; 5 Sydney School of Veterinary Science, Faculty of Science, The University of Sydney, Sydney, Australia

**Keywords:** Sows, Growers, Piglets, Animal welfare, Research for development, Smallholder farmer

## Abstract

Smallholder pig farms are an important feature of pork supply in Asia, and these farming approaches present unique management considerations. This survey was conducted to pilot a welfare assessment protocol and to investigate smallholder pig management and welfare at two sites in Vietnam, one with 55 farms raising predominantly indigenous pigs (the Da Bac site) and the other 55 farms raising predominantly non-indigenous pigs (the Lac Son site). Farmers at both sites kept small numbers of confined pigs, with more keeping sows in Da Bac and some keeping only grower pigs in Lac Son. Via an interview and pig observation, data on 18 welfare measures were recorded for adult pigs, suckling piglets and grower pigs. Pigs on > 90% of farms did not exhibit panting, tail biting, scouring, negative social behaviour, stereotypic behaviour, coughing or lameness demonstrating adequate welfare for these welfare measures. However, all pigs on > 50% of farms had restricted access to drinking water at both sites, and pigs often displayed fear of humans in the Da Bac site. The body condition of most pigs was thin or very thin among the sows on 31% of farms and among the grower pigs on 32% of farms. This pilot proved that the welfare assessment protocol is feasible and provided lessons for its future use in other low-to-middle income countries. It also found that low-cost interventions are urgently needed to address water access and undernutrition in order to improve the welfare of smallholder pigs and the production of these farms.

**Supplementary information:**

The online version contains supplementary material available at 10.1007/s11250-025-04602-5.

## Introduction

Smallholder pig production systems are an important feature of communities in low-to-middle income countries (LMIC) and contribute to the financial and socio-cultural stability of households, particularly in rural areas. The importance of this sector is demonstrated by the marked growth in pig numbers raised by smallholder farmers in countries such as Uganda (FAO Statistical Division 2023; Uganda Bureau Of Statistics 2023) and the substantial proportion of pigs produced on smallholder farms in the Philippines (76% in 2023), despite the adverse impact of African swine fever outbreaks since 2019 (Philippine Statistics Authority 2023). For mixed crop-livestock farming and for peri-urban settings, advantages offered by pigs compared to other livestock include comparatively fast returns, limited land requirements, and utilisation of crop residues, household and restaurant waste as feed (Marshall et al. 2023).

In Vietnam, pork is the most consumed meat, accounting for 65% of per capita meat consumption in 2023, with annual per capita pork consumption expected to rise (OECD 2018). Similar to the Philippines, consumer demand has been predominantly met by pigs raised on smallholder farms, which comprised 77.5% of pig farms in 2011 (General Statistics Office 2012). A pig farm rearing 1–2 sows and producing 4–10 pigs for sale/slaughter per year is designated as a household farm in Vietnam (General Statistics Office of Vietnam 2020). From 2014, government policies have fostered a gradual transition toward larger sized farms with better biosecurity and less environmental impact, and encouraged private investors to establish industrial farms (General Statistics Office of Vietnam 2020; Ministry of Agriculture and Rural Development 2019, Prime Minister of Vietnam. 2020). In 2022, the Vietnam pork industry, comprised of household farms (produce < 50 pigs/year), small-scale farms (produce 50–149 pigs/year), semi-industrial farms (produce 50–1500 pigs/year) and industrial farms (produce > 1500 pigs/year), ranked second in Asia and sixth globally in pork production, with recovery ongoing from losses due to African swine fever outbreaks since 2019 (General Statistics Office of Vietnam 2024, Sharifuzzaman et al. 2024).

Pressure to establish more intensive piggeries to meet growing consumer demand for meat is in potential conflict with animal welfare (Dawkins 2016). While monitoring the welfare of livestock is standard practice on farms in high-income countries, consideration of animal welfare is still emerging across LMIC. Awareness of animal welfare, as per the Five Domains Model (Nutrition, Physical environment, Health, Behavioural interaction, Overall mental state) (Mellor et al. 2020), and motivation to measure it in these countries is being driven by interest to gain access to export markets with animal welfare requirements (Bowles et al. 2005) and emerging domestic consumer concern about the conditions for animals on farm and at slaughter (Miranda-de la Lama et al. 2019; Carnovale et al. 2021; Estévez-Moreno et al. 2022).

In Vietnam, the first animal welfare regulation, the Vietnam Law on Animal Husbandry 2018, includes the provision of feed, water, housing, treatment and prevention of disease, and restriction of trauma, fear and pain for livestock (National Assembly Socialist Republic of Viet Nam 2018). Although 84.3% of pork consumers interviewed in a 2019 survey in Vietnam had not heard of the term “animal welfare”, the majority expressed concern about pig welfare on farm and at slaughter (Le et al. 2022). To date, investigation of pig welfare in Vietnam is limited to one study on pregnant sow welfare in four production systems, including the smallholder pen-based system, smallholder stall-based system, semi-intensive stall-based system, and intensive stall-based system, using 10 animal-based measures of the Welfare Quality® protocol 2009 (Quang Hanh et al. 2024). In relation to smallholder pig systems, while there are calls for knowledge about pig welfare status and for change where welfare is compromised (World Animal Protection et al. 2021), the absence of context appropriate on-farm welfare assessment protocols is limiting research on this topic globally.

Following a scoping review that confirmed this lack of suitable protocols, Oehlers et al. (202*) formulated the preliminary version of a new protocol specifically for application in smallholder pig systems in LMIC by collating and redefining the most relevant welfare measures from various existing protocols. As animal welfare is a multidimensional concept, with better welfare encompassing absence from suffering and the ability to exhibit a high level of biological functioning and positive behavioural experiences, this on-farm welfare assessment protocol included resource-based, management-based and animal-based welfare measures. Designed for the purposes of benchmarking and identifying problem areas, the protocol comprised a questionnaire (15 questions - pig herd demographics, housing, management, biosecurity) and an observation record sheet (23 welfare measures – 7 resource-based, 16 animal-based). In combination, these provide an assessment of pig welfare across the four physical domains of the FDM.

The objectives of this survey were to (1) describe the management and assess the welfare of pigs raised in two contrasting smallholder pig systems in Vietnam, and (2) obtain initial information on farmer knowledge of animal welfare and about farmer access to capital and advice on pig feeding. As the first pilot of the smallholder pig welfare assessment protocol proposed by Oehlers et al. (202*), it also served to evaluate the utility of this protocol and suggest areas for improvement in future investigations of smallholder pig welfare in LMIC.

## Materials and methods

A cross-sectional survey involving on-farm visits was conducted during February 2023 in two districts of Hoa Binh province, northern Vietnam, with contrasting smallholder pig systems.

### Selection of study sites and of household pig farms

Hoa Binh province, a mountainous province in the northwest of Vietnam, was purposely chosen based on the pig population of 0.44 million head in 2022 and the diversity of pig raising including several industrial, 140 semi-industrial farms and a large number of small-scale farms. In relation to the small-scale pig farms, the situation for indigenous pig herds and for non-indigenous pig farms in Hoa Binh province is typical of that present throughout Vietnam.

Of the 10 districts in the province, the two districts with the largest pig populations among districts raising indigenous pigs (2/10) and raising non-indigenous pigs (8/10) were selected, Da Bac (DB) and Lac Son (LS), respectively. Within each selected district, the commune with the highest number of small-scale pig farms was selected in consultation with veterinarians at Hoa Binh Sub-Department of Animal Health. A list of small-scale pig farms in each selected commune was obtained from the commune veterinarian and stratified by herd size: for Da Bac based on number of sows (1–2, 3–5, > 5 sows), and for Lac Son based on total herd size excluding suckling piglets (<5, 5–10, > 10 sows). A proportional random sample of 55 farms by herd size was then selected in each commune. In the event that a selected farm did not participate, a replacement farm was randomly selected from the list.

The sample size was based on a two-proportion comparison, with the assumption that the proportion of farmers aware of animal welfare would differ between the two districts, estimated at 20% and 47% for indigenous and non-indigenous farmers, respectively, along with confidence level of 95% and power of 80% (Dhand and Khatkar 2014). The required sample size was 52 per district, and 55 farms were actually selected per district.

### Engagement of farmer

An invitation to participate in the survey was extended to each selected farm via a phone call made by the commune veterinarian. During the phone call, the purpose of the survey was explained along with participation being voluntary and collected information being managed in a confidential and de-identified manner by the research team only. On the day of farm visit, these points were reiterated by the research team to the household member responsible for pig management and written consent was obtained from this person to participate in the survey and for observation of the pigs (including entry to the pig pen/s) and photographs of the pig housing.

### Questionnaire and observation record sheet

The smallholder pig welfare assessment protocol developed by Oehlers et al. (202*), consisting of a questionnaire and pig observation record sheet, was modified by the research team to suit the smallholder pig raising context at the study sites. The following were deleted: measures of temperature and ammonia level (due absence of indoor pig housing at study sites) and evaluation of parasite burden via faecal egg count and skin scraping (due sample collection and diagnostic evaluation being impractical at the study sites).

The questionnaire administered in this survey consisted of 47 questions (predominantly close-ended questions) across 6 sections: Farmer demographics (11 questions); Pig herd structure (2); Pig housing (8); Pig management (15); Farm biosecurity (6); Sources of capital and advice to improve pig management (3); Understanding of animal welfare (2).

The observation record sheet used in this survey included the 18 measures shown in Table 1. These were recorded separately for each category of pigs present on the farm: Adult pigs (sow, gilt, castrated adult male, boar), Suckling piglets (piglets with sow), Grower pigs (weaners to fatteners). When pigs belonging to one pig category were housed separately then observation of each pig was done and collated results recorded on one record sheet. For a sow with suckling piglets, observation data for the sow was recorded on the Adult pig record sheet and for the piglets on the Suckling piglet record sheet. On the small number of farms with many pens of pigs belonging to one pig category (applied only to grower pigs in Lac Son), one pen was randomly selected for observation.

**Table 1:** Observation record sheet used in a survey of 110 smallholder pig farms in Hoa Binh province, Vietnam in 2022. A = Adult pigs (sow, gilt, boar, castrated male), SP = Suckling piglet, G = Grower pig, *0-10% of pigs observed. 12 measures scored by the researcher observing the pigs from outside the pen without disturbing them (10 minute observation time). 1 measure scored by the researcher observing the pigs as he/she entered the pen and approached the pigs. 5 measures scored by the researcher observing the pigs when the researcher actively disturbed the pigs (10 minute observation time)

Measure/Score allocation	A	SP	G	Instructions
**PANTING** 2 - No pigs are panting* 1 - > 10% but < 50% of pigs are panting 0 - > 50% of pigs are panting				1. Watch breathing/respiratory rate (RR), RR of > 28 breaths/minute for adult and > 55 breaths/minute for piglet is panting. 2. Following this guide, estimate the proportion of pigs panting. 3. Record the corresponding score
**HUDDLING** 2 - No huddling* 1 - > 10% but < 50% of pigs are huddling 0 - > 50% of pigs are huddling				1. Evidence of huddling: if more than 10% of pigs are lying with more than half of their bodies in contact with another pig, then the pigs are huddling. 2. Following this guide, estimate the proportion of pigs huddling. 3. Record the corresponding score
**TAIL BITING** 2 - No evidence of tail biting* 1 - > 10% but < 50% of pigs show evidence of tail biting 0 - > 50% of pigs show evidence of tail biting				1. Evidence of tail biting: fresh blood on the tail, missing tail, swelling and redness of the tail, or wounds fresh or encrusted on the tail. 2. Following this guide, estimate the proportion of pigs affected by tail biting. 3. Record the corresponding score
**SCOURING** 2 - No evidence of scouring* 1 - > 10% but < 50% of pigs show evidence of scouring 0 - > 50% of pigs show evidence of scouring				1. Evidence of scouring: liquid manure visible on their rear or observed passing liquid faeces. 2. Following this guide, estimate the proportion of pigs scouring. 3. Record the corresponding score
**NEGATIVE SOCIAL BEHAVIOUR** 2 - No negative social behaviour* 1 - > 10% but < 50% of pigs exhibiting negative social behaviour 0 - > 50% of pigs exhibiting negative social behaviour				1. Evidence of negative behaviour: biting, charging, tail biting, rough pushing, signs of aggression. But not including play fighting among suckling piglets and weaners. 2. Following this guide, estimate the proportion of pigs exhibiting negative social behaviour. 3. Record the corresponding score
**POSITIVE SOCIAL BEHAVIOUR** 2 - > 50% of pigs exhibiting positive social behaviour 1 - > 10% but < 50% of pigs exhibiting positive social behaviour 0 - No positive social behaviours*				1. Evidence of positive social behaviour: sniffing, gentle nosing, licking, and walking around each other with no flight or aggressive reactions, playing and play fighting among suckling piglets and weaners. 2. Following this guide, estimate the proportion of pigs exhibiting positive social behaviour. 3. Record the corresponding score
**STEREOTYPIC BEHAVIOUR** 2 - No stereotypic behaviour observed* 1 - > 10% but < 50% of pigs exhibiting stereotypic behaviour 0 - > 50% of pigs exhibiting stereotypic behaviour				1. Evidence of stereotypic behaviour: sham chewing (chewing without eating), tongue rolling, teeth grinding (jaw moving without eating), repetitively biting the same bar/trough/drinker, repetitively licking the same spot on the floor. 2. Following this guide, estimate the proportion of pigs exhibiting stereotypic behaviour. 3. Record the corresponding score
**ENVIRONMENT INVESTIGATION** 2 - > 50% of pigs are investigating the environment 1 - > 10% but < 50% of pigs are investigating the environment 0 - No evidence of investigating the environment*				1. Evidence of investigating the environment: sniffing, nosing or licking features in their environment (excluding interaction with other pigs and stereotypic behaviours). 2. Following this guide, estimate the proportion of pigs investigating the environment. 3. Record the corresponding score
**IF ENRICHMENT IS PRESENT** **ENRICHMENT USE** 2 - > 50% of pigs using enrichment 1 - > 10% but < 50% of pigs using enrichment 0 - No pigs using enrichment* 99 – No enrichment provided				1. Evidence of enrichment use: Playing with the enrichment provided 2. Following this guide, estimate the proportion of pigs using enrichment. 3. Record the corresponding score
**WATER SUPPLY** 2 – Free access to water 24/7 0 – Restricted access				1. Evidence of access to water: Presence of water for drinking available to the pigs all the time 2. Record the corresponding score
**DISTANCE TO ACCESS WATER** 2 - < 50 m 1–50-100 m 0 - > 100 m				1. Calculate the distance the pigs have to walk to access water for drinking 2. Record the corresponding score
**FEEDER SPACE** 2 – Adequate space 1 – Inadequate space				1. Evidence of feeder space: Space available for pigs to access feed 2. Observe the feed trough/container and the number of pigs in the pen to determine if there is adequate space for all pigs to access to feed simultaneously when feed is present in the trough/container 3. Record the corresponding score
**FEAR OF HUMANS** 2 - > 50% of pigs allow assessor to touch between their ears without withdrawing 1 - > 50% of pigs withdraw initially when assessor approaches them but allows assessor to touch between the ears 0 - > 50% of pigs do not allow assessor to approach and touch them between the ears and withdraw and remain at a distance				1. Enter the pen and approach the pigs. Attempt to touch ≥ 1 pig between the ears. 2. Evidence of fear of humans: pig/s deliberately move away and remain at a distance, pig/s do not allow the researcher to touch between the ears. 3.Following this guide, estimate the proportion of pigs exhibiting fear of humans. Determine and record the corresponding score
**COUGHING** 2 - No coughing observed* 1 - > 10% but < 50% of pigs coughing 0 - > 50% of pigs coughing	1. 2. 3. Average	1. 2. 3. Average	1. 2. 3. Average	1. Researcher disturbs the pigs so they are moving around in the pen over a 10-minute observation period 2. Count the number of coughs heard in a 3-minute period, repeat this three times and calculate the average of the three counts 3. Evidence of coughing: If the average is > 5 coughs in a 3-minute period, the pigs are coughing 4. Record the corresponding score
**LAMENESS** 2 - No lameness observed, normal gait 1 - > 50% of pigs are lame on at least one leg 0 - > 50% of pigs are non-weight bearing on at least one leg				1. Researcher disturbs the pigs so all the pigs in the pen are standing and moving around 2. Observe their gait and make note if pig/s are walking using all limbs equally 3. Based on observation, estimate the proportion of pigs exhibiting lameness. 4. Record the corresponding score
**BODY CONDITION SCORE** 2 - > 50% of pigs BCS > 2 1 - > 50% of pigs BCS = 2 0 - > 50% of pigs BCS < 2				1. Approach each pig in the pen/pig category and score the body condition using the Model Code of Practice for the Welfare of Animals – Pigs^*^. This involves touching the pig and assessing individual BCS. 2. Based on observation of each pig, estimate the proportion of pigs with BCS > 2, BCS = 2, BCS < 2 3. Record the corresponding score
**SKIN LESIONS** 2 - > 50% of pigs have 4 lesions or less visible 1 - > 50% of pigs have 5–10 lesions visible 0 - > 50% of pigs have > 10 lesions visible				1. Approach each pig in the pen/pig category and observe the whole body for skin lesions, count the number of skin lesions. 2. Based on observation of each pig, estimate the proportion of pigs with ≤ 4 lesions, 5–10 lesions, > 10 lesions 3. Record the corresponding score
**NUMBER OF SICK/INJURED PIGS**				1. Observe all pigs for signs of sickness and injury 2. Count the number of pigs showing any signs of sickness and/or injury (excluding tail biting and skin lesions)

^*^ Model Code of Practice for the Welfare of Animals, Pigs. Third edition. CSIRO. 2008 https://www.publish.csiro.au/ebook/download/pdf/5698

The questionnaire (Supplementary information 1) and observation record sheet (Table 1) were translated to Vietnamese and back-translated to English by two researchers proficient in both languages (6^th^ author and 1^st^ author, respectively) and identified discrepancies in meaning were corrected in the Vietnamese version.

### Data collection

A team of five animal scientists with prior experience in conduct of interviews and on-farm visits met for a training session to become familiar with the questionnaire and to learn the procedure for observation and recording.

A pre-test of the questionnaire and the observation procedure was completed on three farms in Hoa Binh, and subsequently adjustments were made to specific questions and aspects of the observation procedure.

The on-farm visit to each selected household pig farm involved a face-to-face interview and pig observation. First, the interview was conducted at the household residence and completed on average in 60 minutes. Second, the observation record sheet was completed where the pigs were housed by 2 or 3 researchers and observation recording usually completed in 20–45 minutes total depending on herd size, with one researcher completing the observation recording per pig category. The research team wore personal protective equipment (PPE) during conduct of the observations and a strict biosecurity protocol was adhered to when moving from farm-to-farm during data collection to ensure that no pathogen transmission occurred between farms.

### Data management and analysis

Upon conclusion of data collection, the data were entered into purpose-built spreadsheets in Microsoft Excel® 2021. Data cleaning and verification were conducted with checks against the hardcopies when needed. For 10 close-ended questions that recorded continuous data or data with a notable number of other free-text responses, the data were categorised/re-categorised retrospectively.

Standard statistical analyses were performed in RStudio (version 4.4.0) with categorical variables described using frequency tables and continuous variables by mean, median and range. The statistical significance of differences between the two study sites were assessed using Chi square test or Fishers exact test for categorical variables and Wilcoxon test and Kruskal-Wallis test for continuous variables (Petrie and Watson 2013). *P*-value for significance set at < 0.05, otherwise results were considered not statistically significant (NS).

On review of the data for the 18 measures recorded by observation, it was identified that the data on two measures (Enrichment use, Number of sick/injured pigs) were not suitable for inclusion in a sum of measure scores to provide a total welfare score per pig category (adult pigs, suckling piglets, grower pigs). Thus, the total welfare score per pig category was a sum of the scores for 16 measures with a maximum possible total welfare score of 32. Differences in the distribution of each of the 16 welfare measures individually and the total welfare score per pig category between study sites, herd types and housing types were examined for statistical significance by conduct of the Wilcoxon test for study site (2 categories) and the Kruskal-Wallis test for herd type and housing type (each with 3 categories).

## Results

### Farmer demographics

A total of 55 farmers raising small pigs participated at each site (Table 2). Among these farmers, there were fewer female farmers (41.8% DB, 52.7% LS; NS) and less farmers that had completed high school or university (21.8% DB, 41.9% LS; NS) at the Da Bac site. The participating farmers at the Lac Son site were predominantly Muong (94.5%) with livestock raising as the most common primary household income source (41.8%) compared to a mix of Dao (60.0%) and Tay (36.4%) ethnicities at the Da Bac site, where crop production was the most common primary household income source (43.6%) and half of the participants reported a low average monthly income of USD 0–200 (50.9% DB, 23.5% LS; P = 0.016). The farmers at both sites raised pigs for similar purposes though more farmers in Da Bac stated that pigs were raised for family celebrations (38.2% DB, 21.8% LS; P = 0.09) and for gifts (16.4% DB, 3.6% LS; P = 0.06). Virtually all participants stated pigs are kept for household income generation (96.4% DB, 100% LS). While pig disease was the most common major problem encountered in pig raising at both sites (96.4% DB, 76.4% LS; P = 0.005), differences in problems faced between sites were a lack of feed for the pigs in Da Bac (38.2% DB, 16.4%; P = 0.018) and low or fluctuating pig sale price (23.6% DB, 80.0% LS; P < 0.001) along with a rise in commercial pig feed price (0% DB, 20.0% LS; P = 0.001) in Lac Son. The farmers had similar mean years of experience raising pigs between the sites with 20.8 years (median 18, range 3–50) in Da Bac and 17.2 years (median 14, range 1–46) in Lac Son, though the proportion that had raised pigs for ≤ 10 years was higher in Lac Son (21.8% DB, 41.8% LS; NS).

**Table 2:** Demographics of 110 smallholder pig farmers at two districts in Hao Binh province, Vietnam in 2022

Variable	Da Bac (n = 55) Number (%)	Lac Son (n = 55) Number (%)	Overall (n = 110) Number (%)
**Age (years)**			
≤ 35	14 (25.5)	10 (18.2)	24 (21.5)
36-45	14 (25.5)	13 (23.6)	27 (24.5)
46-55	12 (21.8)	16 (29.1)	28 (25.5)
≥ 56	15 (27.3)	16 (29.1)	31 (28.2)
**Gender**			
Female	23 (41.8)	29 (52.7)	52 (47.3)
Male	32 (58.2)	26 (47.3)	58 (52.7)
**Level of education completed**			
None	3 (5.5)	0 (0.0)	3 (2.7)
Primary school (grade 1–5)	17 (30.9)	8 (14.5)	25 (22.7)
Secondary school (grade 6–9)	23 (41.8)	24 (43.6)	47 (42.7)
High school (grade 9–12)	12 (21.8)	20 (36.4)	32 (29.1)
University and above	0 (0.0)	3 (5.5)	3 (2.7)
**Ethnic**			
Kinh	0 (0.0)	2 (3.6)	2 (1.8)
Dao	33^a^ (60.0)	0^b^ (0.0)	33 (30.0)
Muong	2^a^ (3.6)	52^b^ (94.5)	54 (49.1)
Tay	20^a^ (36.4)	1^b^ (1.8)	21 (19.1)
**Source of Income**			
Crop production	24^a^ (43.6)	9^b^ (16.4)	33 (30.0)
Freelancer/casual labourer	14 (25.5)	11 (20.0)	25 (22.7)
Livestock raising	8^a^ (14.5)	23^b^ (41.8)	31 (28.2)
Private business	1 (1.8)	3 (5.5)	4 (3.6)
Salary worker	2 (3.6)	8 (14.5)	10 (9.1)
Other	6 (10.9)	1 (1.8)	7 (6.4)
**Average monthly income**			
0-200 USD	28^a^ (50.9)	13^b^ (23.6)	41 (37.3)
201-500 USD	27 (49.1)	36 (65.5)	63 (57.3)
501-1000 USD	0 (0.0)	6 (10.9)	6 (5.5)
Purpose of raising pig*			
Family celebrations^c^	21 (38.2)	12 (21.8)	33 (30.0)
Communal ceremonies^d^	49 (89.1)	51 (92.7)	100 (90.9)
Household food/consumption	36 (65.5)	35 (63.6)	71 (64.5)
Sell/generate household income	53 (96.4)	55 (100)	108 (98.2)
Gift	9 (16.4)	2 (3.6)	11 (10.0)
Other	1 (1.8)	0 (0.0)	1 (0.9)
**Year of experience raising pigs**			
≤ 10	12 (21.8)	23 (41.8)	35 (31.8)
10 to 19	20 (36.4)	13 (23.6)	33 (30.0)
20 to 29	11 (20.0)	12 (21.8)	23 (20.9)
≥ 30	12 (21.8)	7 (12.7)	19 (17.3)
Major problem faced raising pigs*			
Pig diseases	53^a^ (96.4)	42^b^ (76.4)	95 (86.4)
Lack of capital/money to invest	25 (45.5)	19 (34.5)	44 (40.0)
No market/not easy to sell pigs	6 (10.9)	10 (18.2)	16 (14.5)
Lack of technical knowledge in raising pigs	26 (47.3)	16 (29.1)	42 (38.2)
Lack of feed	21^a^ (38.2)	9^b^ (16.4)	30 (27.3)
Lack of water	2 (3.6)	2 (3.6)	4 (3.6)
High mortality	5 (9.1)	0 (0.0)	5 (4.5)
Poor breed quality	2 (3.6)	1 (1.8)	3 (2.7)
A surge in the price of commercial feed	0^a^ (0.0)	11^b^ (20.0)	11 (10.0)
Pig market price is low or fluctuating	13^a^ (23.6)	44^b^ (80.0)	57 (51.8)
Other	5 (9.1)	1 (1.8)	6 (5.5)

* Multiple choice question

^a, b^ Different characters in the same row means statistically different (P < 0.05)

^c^ Family celebrations such as weddings, funerals, birthdays

^d^ Communal ceremonies such as religious ceremonies, public holidays

### Pig farm demographics and confinement

As determined by site selection, the small-scale pig farms differed between the two sites. In Da Bac, nearly all pig farms raised only indigenous/local breed pigs (53/55, 96.4%) and the mean total herd size (excluding suckling piglets) was 4.7 ± 4.3 pigs (median 3, range 1–20) including a mean number of 1.8 ± 1.0 sows (median 1, range 1–5). In contrast, in Lac Son, the vast majority of pig farms raised only non-indigenous/exotic breed pigs (51/55, 92.7%) and herds were larger in size (total herd size (excluding suckling piglets) mean 17.3 ± 22.2 pigs, median 11, range 1–115; sow number mean 2.8 ± 2.5 sows, median 2, range 1–15)).

On the day of visit, 52 (94.5%) farms had a sow/s in Da Bac, of which 22 also had grower pigs and 15 had grower pigs and boar. In Lac Son, no farms had a boar, and sows were present on only 43 (78.1%) farms with or without grower pigs, while 12 (21.8%) farms had a pig herd composed entirely of grower pigs (Table 3). All pigs on the 55 Lac Son farms were confined in pens and on a single farm some pig pens had a yard (an enclosed area accessible for the pigs to move freely about in), whereas in Da Bac, only 35 (63.6%) farms had all pigs confined in pens without a yard (Table 3). Pen construction and size, the presence of enrichment in pens and pen cleaning are described in Table 3. For the pigs in Da Bac confined in pens with a yard, there was a large variation in yard size with the mean yard area per pig being 32.2 ± 42.2 m^2^ (median 14, range 1–163). Further, two Da Bac farmers referred to suckling piglets in their herd as free-roaming because these small pigs were able to pass through pen/yard sides and wander around their farm/residence.

**Table 3:** Demographics and confinement of 110 smallholder pig farms in two districts of Hao Binh province, Vietnam in 2022

Variable	Da Bac (n = 55) Number (%)	Lac Son (n = 55) Number (%)	Overall (n = 110) Number (%)
**Pig herd type**			
Grower	3^a^ (5.5)	12^b^ (21.8)	15 (13.6)
Sow + Grower	22^a^ (40.0)	36^b^ (65.5)	58 (52.7)
Sow + Grower + Boar	15^a^ (27.3)	0**^b^	15 (13.6)
Sow + Piglet	15 (27.3)	7 (12.7)	22 (20.0)
**Housing type for adult pigs**	*n = 52*	*n = 43*	*n = 95*
Pen/s without yard	38^a^ (73.1)	42^b^ (97.7)	80 (84.2)
Pen/s with yard	11^a^ (21.1)	0.0^b^	11 (11.6)
Pen/s with and without a yard	3 (5.8)	1 (2.3)	4 (4.2)
**Housing type for suckling piglets**	*n = 13*	*n = 18*	*n = 31*
Pen/s without yard	7^a^ (53.8)	18^b^ (100)	25 (80.6)
Pen/s with yard	6^a^ (46.2)	0.0^b^	6 (19.4)
Pen/s with and without a yard	0.0	0.0	0.0
**Housing type for grower pigs**	*n = 35*	*n = 48*	*n = 83*
Pen/s without yard	19^a^ (54.3)	47^b^ (97.9)	66 (79.5)
Pen/s with yard	15^a^ (42.9)	0.0^b^	15 (18.1)
Pen/s with and without a yard	1 (2.8)	1 (2.1)	2 (2.4)
***Pen construction***			
Floor substrate*			
Concrete	51 (92.7)	55 (100)	106 (96.4)
Dirt	5 (9.1)	0 (0.0)	5 (4.5)
Bamboo	2 (3.6)	0 (0.0)	2 (1.8)
Type of pen wall*			
Brick	48^a^ (87.3)	55^b^ (100)	103 (93.6)
Bamboo	13^a^ (23.6)	0^b^ (0.0)	13 (11.8)
Wood	5 (9.1)	0 (0.0)	5 (4.5)
Metal net	6 (10.9)	0 (0.0)	6 (5.5)
Fibro cement	3 (5.5)	0 (0.0)	3 (2.7)
Type of shade*			
Fibro cement	49 (89.1)	55 (100)	104 (94.5)
Palm leaves	7^a^ (12.7)	0^a^ (0.0)	7 (6.4)
Canvas	5 (9.1)	0 (0.0)	5 (4.5)
Corrugated iron	1 (1.8)	0 (0.0)	1 (0.9)
Feeding container material*			
Concrete or brick	38^a^ (74.5)	54^b^ (98.2)	92 (86.8)
Plastic or tyre	20^a^ (39.2)	0^b^ (0.0)	20 (18.9)
Bamboo	3 (5.9)	0 (0.0)	3 (2.8)
Wood	5 (9.8)	0 (0.0)	5 (4.7)
Automatic feeder	0 (0.0)	6 (10.9)	6 (5.7)
**Enrichment present in pen**^**c**^	31 (56.4)	30 (54.5)	61 (55.5)
***Pen area***			
**Per sow**			
Mean ± SD	7.1 ± 3.2	9.4 ± 5	8.1 ± 4.3
Median (range)	7.5 (1–16)	7.9 (2.4–26)	7.5 (1–26)
**Per grower**			
Mean ± SD	5.2 ± 4.1	2.3 ± 2	3.5 ± 3.3
Median (range)	4 (0.2–15)	1.7 (0.5–12)	2.1 (0.2–15)
**Per boar**			
Mean ± SD	7.9 ± 3.9	N/A	7.9 ± 3.9
Median (range)	7.1 (3–16)	N/A	7.1 (3–16)
***Pen cleaning***			
**Frequency**			
Twice a day	12^a^ (21.8)	28^b^ (50.9)	40 (36.4)
Once a day	28 (50.9)	25 (45.5)	53 (48.2)
Twice a week	7 (12.7)	2 (3.6)	9 (8.2)
Once a week	2 (3.6)	0 (0.0)	2 (1.8)
As needed when the pen is dirty	2 (3.6)	0 (0.0)	2 (1.8)
Only when the pen is empty after sale of pigs	4 (7.3)	0 (0.0)	4 (3.6)
Method*			
Wash with water	44 (80.0)	47 (85.5)	91 (82.7)
Sweep/move with broom/palm leaves/stick	39 (70.9)	37 (67.3)	76 (69.1)
Shovel	12 (21.8)	11 (20.0)	23 (20.9)
Other (sprinkle lime)	1 (1.8)	0 (0.0)	1 (0.9)
Disposal of manure/waste from pen*			
Compost	17 (30.9)	23 (41.8)	40 (36.4)
Put in biogas system	3^a^ (5.5)	18^b^ (32.7)	21 (19.1)
Discharge to open area/garden	24 (43.6)	21 (38.2)	45 (40.9)
Contain in a hole	9 (16.4)	3 (5.5)	12 (10.9)
Other^d^	5 (9.1)	3 (5.5)	8 (7.3)

* Multiple choice question

** Farmers use artificial insemination

^a, b^ Different characters in the same row means statistically different (P < 0.05)

^c^ Enrichment included straw (59/61), dry leaves (10/61), discarded clothes (4/61)

^d^ Other included discharge to river, use as fertilizer, storing in a pile

### Pig management

All farmers provided feed to their pigs 2 or 3 times each day (except one farmer in Da Bac) but 20% (11/55) of farmers in Da Bac did not provide water to their pigs and very few farmers at both sites reported that water was available to their pigs all the time (1.8% DB, 10.9% LS; NS, Table 4). The most commonly reported feeds included in pig diets by farmers in Lac Son were commercial feed (98.2%) and rice bran (70.9%) and in Da Bac were rice bran (76.4%), crops grown in garden (72.7%) and household scraps (65.5%). Overall, a greater diversity of feed stuffs were reported by farmers in Da Bac, with at least one quarter reporting use of commercial feed (40.0%), forest vegetable (30.9%) and local industry products (29.1%).

**Table 4:** Feed and water for pigs reported by 110 smallholder pig herds in two districts of Hao Binh province, Vietnam in 2022

Variable	Da Bac (n = 55) Number (%)	Lac Son (n = 55) Number (%)	Overall (n = 110) Number (%)
**Frequency of feeding**			
1 time per day	1 (1.8)	0 (0.0)	1 (0.9)
2 times per day	43^a^ (78.2)	26^b^ (47.3)	69 (62.7)
3 times per day	11^a^ (20.0)	29^b^ (52.7)	40 (36.4)
Ad libitum	0 (0.0)	0 (0.0)	0 (0.0)
**Feeding method**			
On the ground	0 (0.0)	0 (0.0)	0 (0.0)
In container	55 (100)	55 (100)	110 (100)
Feeds given to pigs*			
Commercial feed	22^a^ (40.0)	54^b^ (98.2)	76 (69.1)
Household scraps	36^a^ (65.5)	7^b^ (12.7)	43 (39.1)
Crops in garden	40^a^ (72.7)	16^b^ (29.1)	56 (50.9)
Rice bran	42 (76.4)	39 (70.9)	81 (73.6)
Restaurant scraps	0 (0.0)	0 (0.0)	0 (0.0)
Local industry scraps	16 (29.1)	13 (23.6)	29 (26.4)
Forest vegetable	17^a^ (30.9)	1^b^ (1.8)	18 (16.4)
Crop-by-product	10 (18.2)	5 (9.1)	15 (13.6)
Other^c^	4 (7.3)	8 (14.5)	12 (10.9)
Method used to cook pig feed*			
Firewood	37^a^ (100)	20^b^ (95.2)	57 (98.3)
Gas	0 (0.0)	1 (4.8)	1 (1.7)
Electricity	0 (0.0)	0 (0.0)	0 (0.0)
Biogas	0 (0.0)	3 (14.3)	3 (5.2)
**Provision of water to pigs**			
No**	11^a^ (20.0)	0^b^ (0.0)	11 (10.0)
Yes	44^a^ (80.0)	55^b^ (100)	99 (90.0)
**Water is always available for pigs**			
No	54 (98.2)	49 (89.1)	103 (93.6)
Yes	1 (1.8)	6 (10.9)	7 (6.4)
Source of water given to pigs*			
Spring water/ ground water seepage	23 (52.3)	22 (40.0)	45 (45.5)
Local pond/river	0 (0.0)	0 (0.0)	0 (0.0)
Well on your property	21 (47.7)	23 (41.8)	44 (44.4)
Communal well	1 (2.3)	0 (0.0)	1 (1.0)
Tap	0^a^ (0.0)	14^b^ (25.5)	14 (14.1)

* Multiple choice question

** Water is available in feed

^a, b^ Different characters in the same row means statistically different (P < 0.05)

^c^ Other included corn meal, cassava, corn, rice

Few farmers reported use of standard farm biosecurity practices to prevent pathogen entry (Table 5). Preventive health care for internal parasites was reported by most farmers at both sites (87.3% DB, 81.8% LS; NS) but vaccination was limited to about one third of the pig herds (36.4% DB, 27.3% LS; NS) and external parasite control was not reported (except for 4 farmers at Da Bac).

**Table 5:** Biosecurity and health care practices reported by 110 smallholder pig herds in two districts of Hao Binh province, Vietnam in 2022

Variable	Da Bac (n = 55) Number (%)	Lac Son (n = 55) Number (%)	Overall (n = 110) Number (%)
New pigs are separated for at least one week	1 (1.8)	2 (3.6)	3 (2.7)
Other people are prevented from coming into contact with pigs	4 (7.3)	6 (10.9)	10 (9.1)
Have specific boots to wear in pig pens	0 (0.0)	4 (7.3)	4 (3.6)
Clean boots used for working with pigs	0 (0.0)	4 (7.3)	4 (3.6)
Disinfect boots used for working with pigs	0 (0.0)	2 (3.6)	2 (1.8)
Frequent use of measures to prevent rodents or insects going into pig pens	8 (14.5)	12 (21.8)	20 (18.2)
Vaccination of pigs	20 (36.4)	15 (27.3)	35 (31.8)
Regular deworming of pigs	48 (87.3)	45 (81.8)	93 (84.5)
Regular use of medicine to control scabies/skin disease of pigs	4 (7.3)	0 (0.0)	4 (3.6)
Most common action when pigs get sick			
Ask help from family or friends/neighbours	0 (0.0)	1 (1.8)	1 (0.9)
Called livestock technician/veterinarian	26^a^ (47.3)	44^b^ (80.0)	70 (63.6)
Just leave it	1 (1.8)	0 (0.0)	1 (0.9)
Use medicine for animals that I bought	22 (40.0)	9 (16.4)	31 (28.2)
Use medicine for people that I bought	1 (1.8)	1 (1.8)	2 (1.8)
Use traditional medication for treatment of sick pigs	5 (9.1)	0 (0.0)	5 (4.5)

^a, b^ Different characters in the same row means statistically different (P < 0.05)

Management of sick pigs commonly involved seeking help from a livestock technician or veterinarian in Lac Son (80.0%), whereas farmer action in Da Bac was more evenly split between seeking help from livestock technician/veterinarian (47.3%) and use of medicine or traditional medicine readily accessible to the farmer (50.9%).

### Farmer sources of advice and capital for pig raising

Farmers stated high reliance on local contacts to source advice on pig feeding and on self and local contacts to source money to build/repair pig pens and purchase pig feeds (Supplementary information 2). The dominant sources of advice at both sites were family, close friends and neighbours (81.8% DB, 90.9% LS; NS). Other advice sources were trainings on pig raising (reported by 21.8% of farmers at both sites), and at Lac Son, the internet was listed by 21.8% of farmers and radio or television by 16.4%. At both sites, use of own saved money was the principal source of money for pig pen improvement (83.6% DB, 94.5% LS; NS) and for pig feed purchase (96.4% DB, 76.4% LS; P = 0.005). Mention of external sources was limited with borrowing from the bank for pen costs stated by 18.2% of farmers in Da Bac and purchase of pig feed on credit from feed suppliers by 45.5% of farmers in Lac Son.

### Farmer knowledge of animal welfare

There was virtually no knowledge of the formal term ‘animal welfare’, with only one farmer at each site reporting that he/she had heard of animal welfare (Online Resource 2). However, there was substantial interest to know about animal welfare in a more formalised way (87.3% DB, 72.7% LS; NS).

### Pig welfare

Based on researcher observation on the day of visit, the distribution of scores for each of 17 welfare measures (excluding sick/injured pigs) are shown for adult pigs, suckling piglets and grower pigs in Tables 6, 7, 8, respectively. Scores of 2 (the highest possible score) were recorded on > 90% of participating farms for adult pigs, suckling piglets and grower pigs for 8 (47.0%) of these 17 welfare measures (panting, tail biting, scouring, negative social behaviour, stereotypic behaviour, coughing, lameness, distance to access water). Furthermore, no or very few pigs across the 110 farms were observed to be panting or coughing, and none had to walk ≥ 50 metres to access drinking water.

**Table 6:**
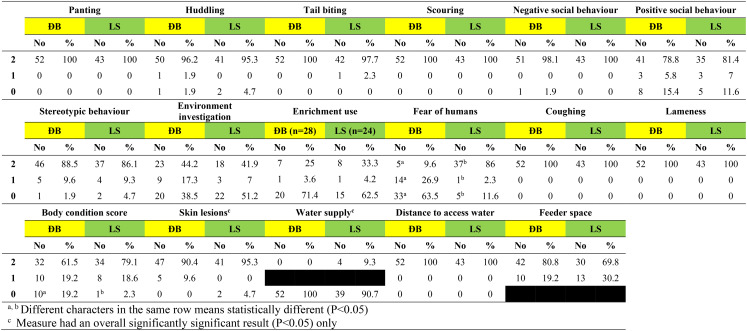
Scores for 17 welfare measures based on observation of adult pigs on 110 smallholder pig farms in two districts of Hao Binh province, Vietnam in 2022. On the day of visit adult pigs were present on 52 farms in Da Bac and 43 farms in Lac Son

**Table 7:**
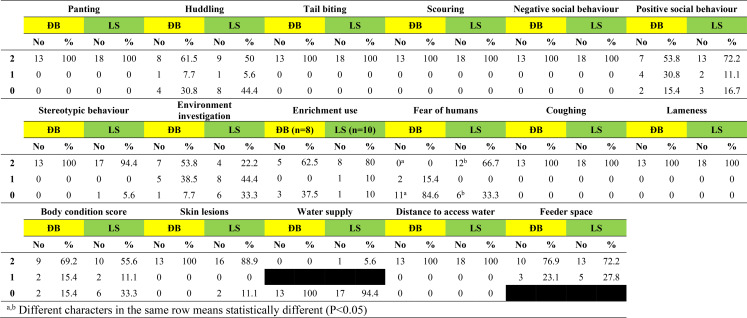
Scores for 17 welfare measures based on observation of suckling piglets on 110 smallholder pig farms in two districts of Hao Binh province, Vietnam in 2022. On the day of visit, suckling piglets were present on 13 farms in Da Bac and 18 farms in Lac Son

**Table 8:**
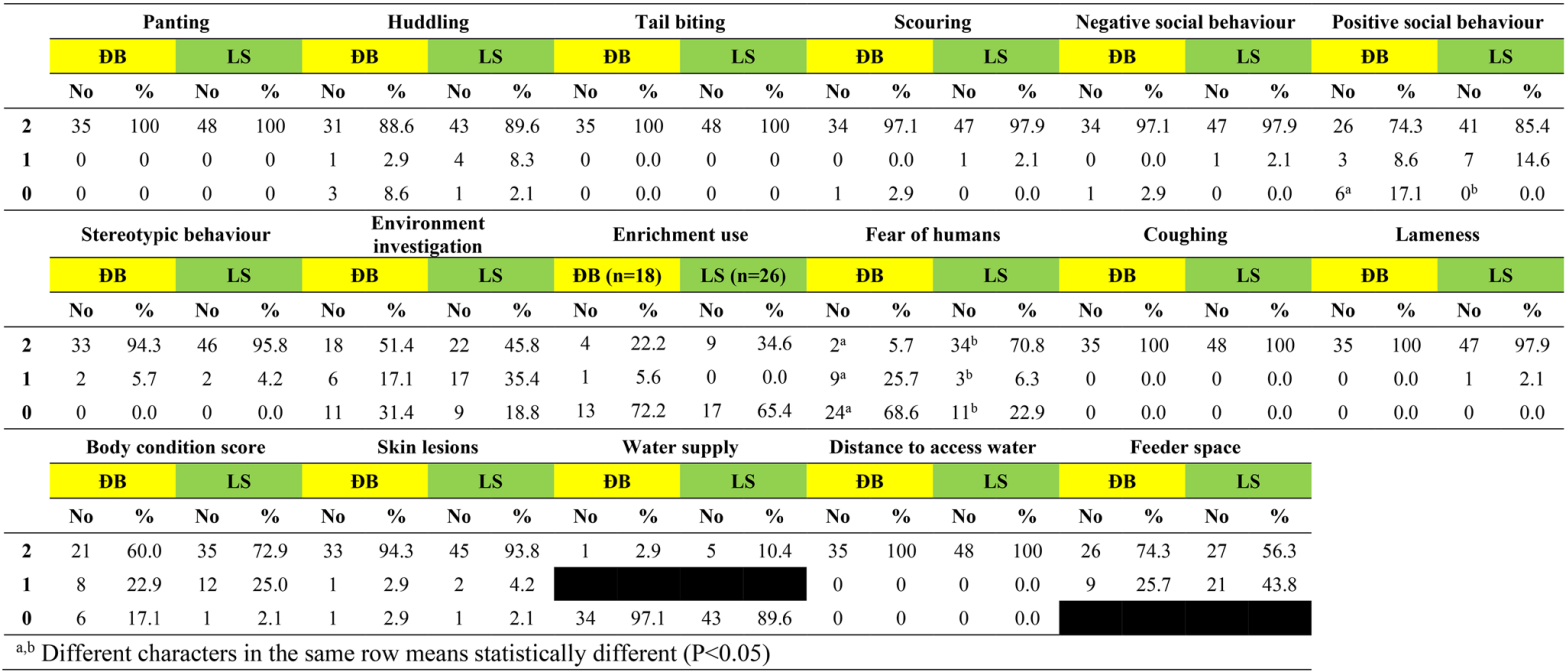
Scores for 17 welfare measures based on observation of grower pigs on 110 smallholder pig farms in two districts of Hao Binh province, Vietnam in 2022. On the day of visit, grower pigs were present on 35 farms in Da Bac and 48 farms in Lac Son

The two welfare measures with scores of 0 (the lowest possible score) recorded on > 50% of participating farms for adult pigs, suckling piglets and grower pigs were fear of humans at the Da Bac site only, and water supply at both Da Bac and Lac Son sites.

Enrichment was provided in pens on just over half the farms in each district for adult pigs, for suckling piglets and for grower pigs, with the lowest proportion of farms providing enrichment being 52.8% for grower pigs in Da Bac and the highest being 61.5% for suckling piglets in Lac Son. Use of the enrichment by adult pigs and by grower pigs was observed on about one third of farms where it was provided (scores 1&2 in Tables 6 & 8). Observed use of enrichment by piglets was higher at 62.5% of farms in Da Bac and 90% of farms in Lac Son (scores 1&2 in Table 7).

Only three farms had pigs with observable signs of sickness or injury. These were 1 farm in Da Bac (where the 2 sows and 2 of 3 grower pigs were recorded as sick/injured) and 2 farms in Lac Son (where 1/66 grower pigs and 4/10 grower pigs were recorded as sick/injured).

**Fig. 1:**
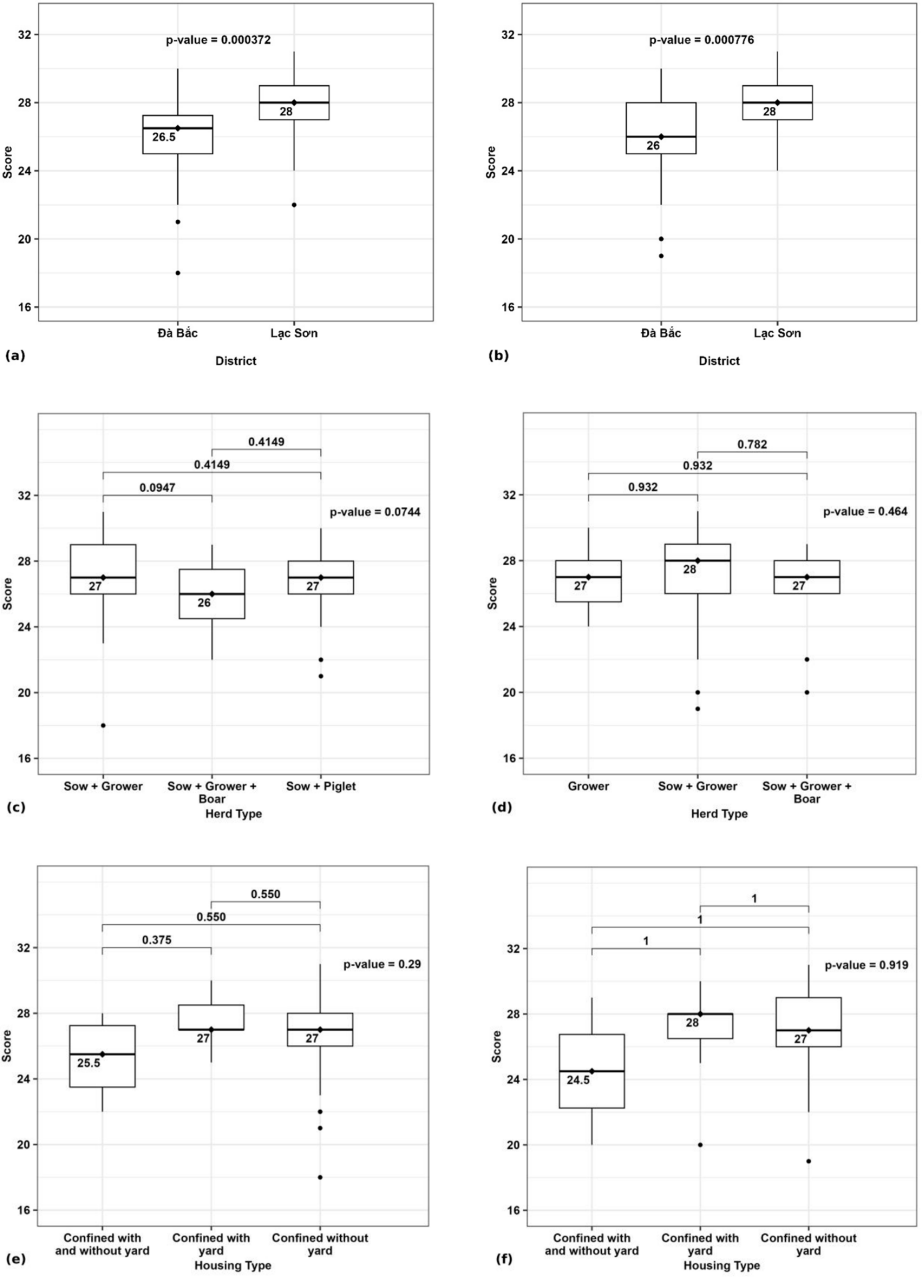
Distribution of total welfare score for adult pigs (52 farms in Da Bac district, 43 farms in Lac Son district) and for grower pigs (35 farms in Da Bac district, 48 farms in Lac Son district) on smallholder pig farms **a**) Adult pigs by district **b**) Grower pigs by district **c**) Adult pigs by herd type **d**) Grower pigs by herd type **e**) Adult pigs by housing type **f**) Grower pig by housing type

The median total welfare scores based on 16 welfare measures per pig category on the study farms was 26.5 (range 18–30) for adult pigs, 26 (range 19–30) for grower pigs and 26 (range 21–28) for suckling piglets in Da Bac and 28 (range 22–31) for adult pigs, 28 (range 24–31) for grower pigs and 26 (range 22–30) for suckling piglets in Lac Son. The total welfare scores across farms in Lac Son were significantly higher than in Da Bac for adult pigs (P = 0.0004) and for grower pigs (P = 0.0007, Fig. 1a, 1b). Comparison of total welfare score for adult pigs between herd types showed a lower score for farms with sows, growers and boar (P = 0.07, Fig. 1c), but this difference was not statistically significant and there were no significant differences in total welfare score between housing types (Fig. 1e, 1 f).

## Discussion

Given the considerable proportion of pigs raised by smallholders across LMIC, and in recognition of their ongoing contribution to pork supply in these countries for the foreseeable future (Sharifuzzaman et al. 2024), the welfare of smallholder pigs deserves attention. This survey was conducted to pilot a welfare assessment protocol proposed for use on smallholder pig farms and it has provided the first whole herd assessment of smallholder pig welfare in Vietnam, and one of the first globally. As such, it provides important learning on the design for a protocol to assess pig welfare on smallholder farms with their diverse systems of pig management and advances knowledge of pig welfare in smallholder contexts for the Asia-Pacific region. Further, this work is a timely contribution to the international literature with the heightened awareness of the benefits that improvements to animal welfare provide, including to achievement of the United Nations Sustainable Development Goals (Doyle et al. 2021).

To investigate pig welfare on the two prominent types of small-scale pig farms in Vietnam, this study was purposively conducted in the two districts of Hoa Binh province with the largest populations of indigenous pigs and non-indigenous pigs, Da Bac and Lac Son, respectively. According to the internationally accepted categorisation of pig production systems, both farm types are examples of small-scale confined pig production, which is a common production system with diverse presentations across LMIC (Food and Agriculture Organization of the United Nations et al. 2010). In this survey, the contrast in dominant pig breed on small-scale farms at each site was accompanied by differences in pig herd structure and some aspects of management. While all farmers at both sites kept pigs for household income generation, a difference in target market was reflected in the smaller-size breeder dominant herds to produce premium priced young pigs for the specialised Da Bac pig market compared to the larger-size grower dominant herds of Lac Son servicing the province pork market (Le et al. 2016).

Overall, total welfare scores based on 16 welfare measures for adult pigs and grower pigs were higher than anticipated based on general knowledge of resource constraints in smallholder pig systems, with median scores of 26–26.5/32 (scores of 81–83%) for Da Bac and 28/32 (score of 88%) for Lac Son, and across all study farms the number with a total score of ≤ 25/32 (score of ≤ 78%) was only 21/95 (22%) farms with adult pigs and 16/83 (19%) farms with grower pigs (Fig. 1). It is notable that total welfare scores for these two pig categories were significantly higher for the Lac Son site, with a number of confounding factors likely to be influencing this finding. At the Da Bac site, the pigs raised under traditional management are purchased at a premium price linked to the cultural status of these pigs and preference for pigs raised on a ‘natural’ diet (Muth et al. 2017a, 2017b). This traditional management that provides pigs with more freedom, reduces the frequency of close interaction with people, which may be a contributor to the significantly poorer scores for fear of humans across all pig categories at the Da Bac site, however these differences are confounded by breed differences, which may also impact behaviour. Pigs in Da Bac also were more often housed in pens with a yard, which gave them simply a greater distance to retreat from and then move toward the assessor during the timed “fear of humans” test. Published comparisons of the same breed pigs in indoor and outdoor systems did not find differences in responses to humans, and piglets raised in outdoor systems have been described as more ‘calm/passive’, whereas pigs raised indoors were described as ‘playful/inquisitive’ (Temple et al. 2011; Lau et al. 2015).

The provision of water emerged as a common risk for pig welfare. There were only three farms (1 DB, 2 LS) where all categories of pigs on-farm had scores of 2 for the welfare measures of water supply (2 = Free access to water 24/7). Water was the extremely limited resource across the surveyed farms, with water supply recorded as restricted (scored as 0) on 107 farms. Adequate water to drink is necessary for optimum pig production and welfare, with insufficient water reducing sow milk production contributing to piglet losses and slowing growth of grower pigs. Daily water requirement varies between pig breeds and categories, the type of feedstuffs and climatic conditions, and these need to be considered when published guidelines are referred to for daily water requirement (Patience 2012). Restricted access to water remains common for confined smallholder pigs throughout LMIC, being limited to water in the feed ration given once or twice per day on a high percentage of farms in Laos (Phengsavanh et al. 2010) and Tanzania (Braae et al. 2016). For settings where water is freely available for confined pigs, the cleanliness of the water can be a problem, as was found for 49% of farms in central Uganda where the dirty water was implicated with disease occurrence, particularly diarrhoea (Dione et al. 2022). For free-roaming pigs, that source all/most of their food and water, water scarcity during dry periods of the year means not only that access to water is limited but also that pigs may have to walk long distances to source water (Chilundo et al. 2020; De Almeida et al. 2021). In communities with sufficient water for household needs, poor water provision for confined pigs can be overcome through use of nipple drinkers (Barnes et al. 2020).

There were only three farms (1 DB, 2 LS) where all categories of pigs on-farm had scores of 2 for the welfare measures of water supply (2 = Free access to water 24/7) and body condition score (2 = > 50% of pigs with BCS > 2). These two welfare measures reflect access to the essential resources of water and food, meaning that the vast majority of participating farmers are struggling to meet the basic needs of their pigs. Body condition score (BCS), an observed measure with a standard 5-score system, reflects nutrition and health status. When animals are healthy, including no/few internal parasites, BCS is determined by the adequacy of feed intake to meet maintenance and production needs. For the surveyed adult pigs and grower pigs, the fact that the majority were thin (on 19% and 24% farms, respectively) or the majority were very thin/emaciated (on 12% and 8% farms, respectively) (Tables 6 & 8) demonstrates severe and sustained nutritional deficiency on these farms. Skinny pigs given imbalanced diets ± less than adequate amounts of feed (ie underfeeding) are common in smallholder pig herds. Dione et al. (2022), recently found > 90% of pigs in all pig categories with BCS 1–2 out of 5 on smallholder farms with penned pigs in central Uganda (Dione et al. 2022). Penned pigs, totally reliant on their owner for food, are particularly vulnerable to undernutrition when feed sources are limited due to dry seasonal conditions or low household income prevents feed purchase. A comparison of free-range pigs and penned pigs in Tanzania found notably more pigs with poor body condition among the penned pigs, along with welfare impacts of poor housing such as no shade for the > 90% of white skinned exotic cross-bred penned pigs (Braae et al. 2016). The finding that significantly more farms in Da Bac had emaciated pigs than in Lac Son points to a more complex interplay of factors impacting nutrition than extent of confinement alone. Feeding of the indigenous pigs in Da Bac is more labour intensive and obtaining adequate amounts of feed was reported to be a problem, with diets comprised mostly of locally sourced rice bran and hand gathered and cooked feed stuffs such as garden crops and forest vegetable. By comparison, reliance on purchased commercial feed and on rice bran (some of which is purchased) to comprise some/all of the pig diet was commonplace in Lac Son and reflected in 20% of farmers reporting rises in commercial feed price as a problem; so while this may be contributing to better BCS of pigs, it comes with inherent risks for ongoing supply and sustainability.

In this study, we also considered feeder space as potentially another contributor to undernutrition, and inadequate feeder space was present on 33 farms. Concern about competition for feed due to insufficient feed trough space adding to underfeeding for smaller and weaker pigs, has also been reported on smallholder pig farms in rural northern Laos (Phengsavanh et al. 2010). Preventive health care is also a known contributor to BCS, however whilst most pig farmers reported regular deworming of pigs, we did not perform faecal egg counts to evaluate internal parasite burden. Even with consideration of similarities and differences in pig feeding, confinement, preventive health care and veterinary services between study sites, the source of the poor BCS is difficult to identify and is likely multifactorial. This is a good example of the complexities of welfare assessment in diverse farming systems, and underlines the importance of capturing data on resource-based measures along with animal-based measures to assist overall understanding of constraints on animal welfare.

The welfare of livestock is highly dependent on farmer knowledge and attitude, which along with the available resources, determine husbandry practices. The foundation for action, knowledge of the formal term of ‘animal welfare’, was virtually non-existent among the surveyed farmers though many stated interest to learn about it. Knowledge of formal term of animal welfare may be important to foster common understanding, appreciate relevant policy issues, and facilitate dialogue around the issue. It does not reflect a lack of awareness of welfare needs and challenges of an owner’s animals, however. A more exploratory approach to inquiry in this survey may have likely revealed knowledge of pig welfare. Investigation using discursive methods identified relevant knowledge among half of small-scale mixed crop-livestock farmers in Ethiopia (Alemayehu et al. 2022). In Kenya, over 70% of small-scale broiler famers were aware of animal welfare, and 80–90% perceived good feeding, good health and suitable behaviour to be indicators of good welfare (Yensuk et al. 2022). As the purpose for animal keeping and the extent of close interaction with animals influence recognition of poor welfare and concern about it, differences in farmer knowledge may even have been elucidated between study sites. External sources, including media, hatcheries, agrovet stores and government extension agents, were reported as important sources of information on broiler welfare by the Kenyan farmers. In this pilot survey, the heavy reliance on self and local networks for advice, with external assistance limited to feed suppliers in Lac Son, may be restricting advances for pig management and welfare.

This pilot, using a modified version of the preliminary welfare assessment protocol for smallholder pig farms Oehlers et al. (202*), has provided some lessons for on-farm welfare assessment on smallholder pig farms in LMIC. These lessons relate to the measures for inclusion in the assessment and method for measurement. First, farm location and pig housing type will determine the relevance for inclusion of measures such as ammonia and temperature, which were excluded for this pilot in the absence of indoor housing, and instead aspects of pen construction (type of wall and of shade) were used to gauge climatic exposure. These also determine the feasibility of certain health measures, which in this pilot were limited to observed measures and farmer recall because there were no laboratory facilities in close proximity to the study site for parasitology diagnostics on pig samples. Further, conditional inclusion of distance to access water, only for pigs that have 24/7 access to water, would more fairly reflect the real benefit of close proximity to water. Second, assessment of welfare measures at the pig category level means that the actual percentage of animals with a zero score for health and behaviour measures was not recorded. Furthermore, the score categories were designated arbitrarily and for a pig category with a score of 1, though some welfare-compromised animals are present (for example, BCS of 1 means > 50% of pigs are thin with BCS = 2), a requirement for action is not flagged. Third, focus on total welfare score based on 16 measures somewhat downplayed critical issues needing immediate intervention, such as water access and way/s it can be provided on-farm to improve pig welfare. Use of non-weighted additive scoring contributed to this by attributing equal influence on overall welfare status to each of the 16 measures. Although we acknowledge the 16 measures are not all of equal influence, weighting of measures would complicate score calculation and may prevent full completion of the assessment on-farm. Thus, refinement by inclusion of measure weightings based on expert elicitation may reduce feasibility of protocol application in low-resource settings, although a mobile phone application has potential in the future. An alternative approach would be to designate a subset of most influential measures for individual consideration, for example as critical measures requiring immediate attention, as suggested by Oehlers et al. (202*) but not implemented in this pilot. While weighting would address the scoring outcomes for benchmarking, it is not necessary for the tool to be used for continuous improvement. The tool is viable as it is to identify where improvements need to be made; weighting is not necessary to prioritise where improvements should be made, and in smallholder settings, prioritisation for improvements comes down to how implementable solutions are.

Of the two other comprehensive studies of smallholder pig welfare, conducted during the same time period as our research (2021–23), both also used a protocol combining an interview and observations (Dione et al. 2022; Rhea et al. 2023). But Dione et al. (2022) implemented alternate approaches that offer potential solutions for some issues encountered in this pilot. These were individual observation of pigs along with group observations to report proportions of pigs for specific health measures and observation of a person familiar to the pigs entering the pen to record fear of humans (Dione et al. 2022).

Whilst the conduct of this pilot in one province may be considered a study limitation, reducing the ability to generalise findings, we consider that the two sites in Hoa Binh province are representative of the management on small-scale farms raising indigenous and non-indigenous pigs across Vietnam. Indigenous pigs are well known to be raised in the more remote, mountainous areas of Vietnam with non-indigenous pigs in rural areas raised by small-scale farmers located near towns to supply provincial demand for pork (Lemke et al. 2008; Huyen et al. 2019). On-farm assessment on a single occasion and by a team of animal scientists (not certified welfare assessors) wearing PPE (disposable overall with hood, googles, gloves, boots) has potentially led to under- or over-estimation of the individual welfare measures as pigs instinctively hide signs of illness when fearful, for example, and also were more hesitant to approach the researcher than normally when people enter their pen. This may in part explain the unexpected absence of sick/injured pigs across the vast majority of study farms. In central Uganda, a similarly trained research team observed very low percentages of pigs across 270 smallholder pig farms with visible signs of injury (the maximum being 3.4% of pigs with shoulder sores), however, visible signs of diarrhoea/scouring were recorded for a higher percentage, being 9.4% of the observed pigs (Dione et al. 2022). Length of observation time was similar in both studies. While short observation times are acknowledged as a reason for misclassification of a measure in some instances, and the observation period here was shorter than the Welfare Quality® protocol 2009 (Welfare Quality® 2009) for example, we believe the duration of time for observations was sufficient. Pigs in smallholder settings are more exposed to humans, and the much smaller herd size are just two aspects of the farm context leading to the use of shorter observation periods in this protocol.

In conclusion, this pilot survey provides the first report of animal welfare on smallholder farms raising indigenous and non-indigenous pigs in Vietnam and reflections on the suitability of the welfare assessment protocol for use on such farms. Aspects of pig welfare found to be compromised at both study sites that demand immediate attention are access to drinking water and undernutrition. Low-cost improvements to pen structure on farms with exotic pigs to reduce heat/temperature stress are also recommended. The affective state of pigs observed in this survey was overall positive as very few farms had pigs displaying stereotypies or negative social behaviours, though pigs would not permit the researcher to approach them on many farms raising indigenous pigs. The design of simple interventions utilising locally available resources is recommended to address the identified constraints on pig welfare, and these changes will likewise lead to improvements in farm productivity and profitability. Further refinement of the piloted pig welfare assessment protocol will produce a context-appropriate tool for use in the evaluation of such interventions.

## Electronic supplementary material

Below is the link to the electronic supplementary material.

Supplementary Material 1

Supplementary Material 2

## Data Availability

The datasets generated and analysed during the pilot survey are not publicly available but are available from the corresponding author on reasonable request.
