# Supplementary material for: Assessing the welfare of smallholder pigs in Vietnam using a pilot protocol
Source: Trop Anim Health Prod. 2025 Sep 23;57(8):392. doi: 10.1007/s11250-025-04602-5 (PMC12457531; doi:10.1007/s11250-025-04602-5)
Supplement: Supplementary file 1 — Supplementary Material 1 [file 11250_2025_4602_MOESM1_ESM.docx]

**SUPPLEMENTARY INFORMATION 1**

**Questionnaire**

**Pilot of Pig Welfare Assessment Protocol in Vietnamese smallholder pig systems**

**INFORMED CONSENT**

**1. Study information:** *researcher to inform participant*

Good morning, I am …………………………………………., a researcher at the Livestock System and Environment Research Department, National Institute of Animal Science. We are conducting this survey amongst small household pig farms on pig welfare. This survey aims to determine current management and condition of pigs and evaluate pig welfare at farm and propose measures to improve pig welfare in Vietnam.

Your participation in this study is entirely voluntary and the duration of interview and observation of your pigs will be about 45 minutes and 30 minutes for the farm observation. You can refuse to answer any questions which you consider as sensitive information or invalid. Your personal information, the information that you provide, and the photographs of your pigs will only serve for study’s purpose and be kept confidential.

**2. Informed consent:** *for participant to read and sign*

I ......................................................., being over 18 years old, hereby consent to participate in research “*Pilot of Pig Welfare Framework in Vietnamese smallholder pig systems*”. I have been given information about the objectives of the research. The details of procedures and any risks have been explained to my satisfaction.

I agree to participate in the research and give my information related to the research objectives on my family socio-economic characteristics, including education, gender, income, pig farming experience, management, knowledge, attitude and practices, as well as animal welfare. I understand that I may not directly benefit from taking part in this research. I am free to withdraw from the project at any time and this will not affect me now or in the future.

I have been informed that, while the information gained in this study will be published, I will not be identified, and individual information will not be divulged.

Do you agree to participate in this study? **1- Yes-/ 2- No**

Do you agree to photographs being taken of your pigs? **1- Yes-/ 2- No**

Participant’s name : ………………………….

Signature/thumb print : ………………………….

Date (dd/mm/yyyy) : ………………………….

**QUESTIONNAIRE (for Interview with the pig farmer)**

**A. Demographics of Farmer**

1. Name of farmer…………………………………………………

2. Location of farm:

a. Village: …………………… b. Commune: ……………………………….

c. District……………………. d. Optional of using GPS/GPS id…….…….

3. Age: ____________years old

4. Gender:

□ Male

□ Female

5. Education – Last level of school attended?

□ Illiterate (never go to school)

□ Primary School

□ Secondary School

□ High School

□ University and above

6. Ethnic group – Last level of school attended?

□ Tay

□ Muong

□ Kinh

□ Dao

□ Other (specify)…………………………

7. What is your main/primary income source for household?

□ Livestock raising

□ Crop/planting

□ Private business

□ Worker

□ Freelancer

□ Jobless

□ Other (specify)…………………………

8. What is your average family income per month?

□ 0-200 USD

□ 201-500 USD

□ 501-1000 USD

□ 1001 USD above

□ Don’t know

9. What is your purpose/reason for raising pigs? *(Tick all applicable)*

□ To sell/generate household income

□ Utilize feed from kitchen/agriculture by-products

□ For household food/consumption

□ Party/celebration/festivities - Family event, e.g., wedding, funeral, birthday

□ Traditional and modern ceremonies - Religious or community ceremony

□ Gift

□ Other (specify)…………………………

10. Experienced year of raising pigs: ______________ years

11. What are the major problems faced with raising pigs? *(Tick all applicable)*

□ Pig diseases (e.g., ASF, FMD, PRRS…)

□ Lack of capital/money to invest

□ Low market price

□ No market/not easy to sell pigs

□ Lack of technical knowledge in raising pigs

□ Lack of feed

□ Lack of water

□ High mortality

□ Breed quality (in-breeding, limited access to good quality pig breed for new stock)

□ Other (specify)…………………………

**B. Pig herd structure & Housing/confinement on day of farm visit**

1. What breed of pigs do you keep? and **the number of pigs in herd** on day of visit in each breed category.

| Local/native breed | Cross breed  (a pig with mix of native and exotic breed) | Exotic/commercial |
| --- | --- | --- |
|  |  |  |

2. Per pig type: How many pigs do you have? and How are they kept?

| **Pig type** | **Total number** | **Number of pigs per housing type** | | |
| --- | --- | --- | --- | --- |
|  |  | **Confined**  **(24/7 in pen)** | **Semi-confined*** | **Free roaming**  **24/7** |
| a. Boar used for breeding |  |  |  |  |
| b. Castrated male |  |  |  |  |
| *Local/native breed* |  |  |  |  |
| *Cross breed* |  |  |  |  |
| *Exotic/commercial* |  |  |  |  |
| c. Sow |  |  |  |  |
| d. Piglets with sow |  |  |  |  |
| e. Grower pigs |  |  |  |  |

* 2f. For semi-confined, how many hours per day are the pigs free roaming? _________ hrs

**For penned pigs: asking following questions**

Note: if all pig types are free roaming, no pen => then SKIP questions 3, 4 and 5 below

3. How frequently do you clean your pig pen? *(Tick one)*

- Twice a day
- Once a day
- Twice a week
- Once a week
- Twice a month
- Once a month
- Never, only do one after selling all pigs
- Not sure because it depends how dirty is the pen

4. How do you clean the pig pen? *(Tick all applicable)*

- Removal using broom or palm leaves or stick
- Wash with water
- Other – Please specify ________________________________________

5. What do you often do with the manure/waste removed from pig pens? *(Tick one)*

- Compose
- Put in biogas system
- Discharge to open area/garden
- Other (specify) ______________________________________________

**C. PIG MANAGEMENT – Feed, Water, Preventive health, Biosecurity**

**C1. FEED**

1. Do you give feed to your pigs?

- Yes
- No => SKIP to C2

2. How many times per day do you give feed to the pigs?

- One time per day
- 2 times per day
- 3 times per day
- Ad libitum–confined pigs with automatic feeder so feed is always available to the pigs
- Other (specify) _________________________________________

3. What method of feeding?

- On the ground
- In container, please describe ________________________________
- Other (specify) _________________________________________

4. What do you normally feed to your pigs? *(Tick all applicable)*

⬜ Commercial feed

⬜ Household scraps

⬜ Crops in garden: cassava, papaya, banana, sweet potatoes, etc

⬜ Rice bran

⬜ Restaurant scraps

⬜ Local industry scraps: tofu and wine rice by-product

⬜ Other (specify) _____________________________

5. What did you feed to your pigs yesterday?

For 2 Sows and 2 Grower pigs as examples: List diet components

| Sow ID | Repro status | (Use code from question B2) | Of these diet components, which ones were bought for use as pig feed? |
| --- | --- | --- | --- |
| #1 |  |  |  |
| #2 |  |  |  |

| Grower pig | Age (m) | (Use code from question B2) | Of these diet components, which ones were bought for use as pig feed? |
| --- | --- | --- | --- |
| #1 |  |  |  |
| #2 |  |  |  |

6. Does the food supply for pigs vary through the year?

⬜ Yes

⬜ No => SKIP to 7

**If YES**

a. Which months have a plentiful supply of food for pigs? _________________________

b. Which months is food scarce for pigs? _______________________________________

7. Do you cook the food you fed to your pigs?

⬜ Yes

⬜ No => SKIP to C2

**If YES**, how do you often cook it? *(Tick one)*

⬜ Firewood

⬜ Gas

⬜ Electricity

⬜ Other (specify) ___________________

**C2. WATER**

1. Do you provide water to drink to your pigs?

⬜ Yes

⬜ No

**If NO**, why do you not provide drinking water to your pigs?

________________________________________________________________________

**If YES**, where do you get the water from for your pigs? *(Tick all applicable)*

⬜ Spring/water seed

⬜ Local pond/river

⬜ Well on your property

⬜ Communal well

⬜ Tap

⬜ Other (specify) ____________________________________

2. Is always water available to your pigs?

⬜ Yes

⬜ No

**C3. PREVENTIVE PIG HEALTH**

1. Do you vaccinate your pigs?

⬜ Yes

⬜ No => SKIP to 4

2. If yes, what vaccines are given to your pigs?

_______________________________

_______________________________

_______________________________

_______________________________

3. What is your primary source of vaccination/advice on vaccination? *(tick one most apply)*

⬜ Own knowledge

⬜ Government vet

⬜ Private veterinarian

⬜ Other AH/Livestock worker

⬜ Other pig farmer

⬜ Other (specify) _____________________________

4. Do you regularly give any medicine for worm treatment to your pigs? *(Tick all applicable)*

- Yes
- No
- Depends government program

**If YES**, what medicine is used? ________________________________________

5. Do you regularly provide any medicine to control scabies or any skin diseases in your pigs?

- Yes
- No

**If YES**, what medicine is used? ________________________________________

6. What will you do if your pig is sick? *(Tick one)*

⬜ Just leave it

⬜ Called livestock technician/veterinarian

⬜ Use medicine for animals that I bought

⬜ Use medicine for people that I bought

⬜ Use traditional medication for treatment of sick pigs

⬜ Ask help from family or friend/neighbor

⬜ Sell the pig/s

⬜ Other (specify) _____________________________

**C4. FARM BIOSECURITY**

1. Do you keep new pigs coming into your herd separated from your pigs for at least 1 week to make sure they are not sick before mixing with your pigs?

- Yes
- No

2. Do you prevent other people from having contact with your pigs (e.g., not allowing visitors to enter the pig pen)?

- Yes
- No

**If YES**, how do you stop people from having contact with your pigs? ________________________________________________________________________

3. Do you have boots that are specifically to wear in pig pens?

- Yes
- No

4. Do you clear and disinfect boots used for working with the pigs?

- Yes
- No

5. Do you feed kitchen or restaurant waste to your pigs?

- Yes
- No

6. Do you often use measures to prevent/catch rodents or insect go into your pig pen?

- Yes
- No

**D. SOURCES OF CAPITAL & ADVICE FOR IMPROVEMENT TO PIG MGT**

1. If you want to build or repair a pig pen, where would you get the money to do this?

⬜ Use saved money

⬜ Borrow from family or friend

⬜ Borrow from bank

⬜ Access from farmer cooperative

⬜ Other (specify) _____________________________

2. If you want to give more feed to your pigs to help them grow more /to improve their health, how would you get the money to buy some feed for your pigs?

⬜ Use saved money

⬜ Borrow from family or friend

⬜ Borrow from bank

⬜ Access from farmer cooperative

⬜ Other (specify) _____________________________

3. Where do you get information/advice on how to improve the feeding of your pigs?

⬜ Family and close friends

⬜ Neighbours

⬜ Community leader

⬜ Government officer/veterinarian

⬜ Radio or television

⬜ Internet

⬜ Trainings on pig raising

⬜ Other (specify) _____________________________

**E. UNDERSTANDING ABOUT ANIMAL WELFARE**

1. Have you ever heard about animal welfare term?

- Yes
- No

**If YES**, what does animal welfare mean to you? _______________________________________________________________________________________________­­­­­­­­­­­­­­­­­_________________________________________________

2. Do you want to know about animal welfare?

- Yes
- No
- Don’t know

**BY OBSERVATION NOT BASED ON INTERVIEW WITH FARMER**

**F. PIG PEN INFORMATION AND ARRANGEMENT**

1. List floor substrate: ………………………………………………………………………….

2. List type of pen walls: ………………………………………………………………………

3. List type of shade (e.g., type of roof; shaded by trees) and estimated percentage of pen that is shaded: ………………………………………………………………………………………

…………………………………………………………………………………………………..

4. List types of enrichment substrate present in pen (eg straw, foliage, wood, logs, rope, rubber sheets, paper, cardboard; or toys (large balls, boxes)).

…………………………………………………………………………………………………..

5. **Drawing/Map of the pig pens** for sows; for growers; for boars

For each pen:

- Draw the pen and list the following on diagram

- Measure length of pen sides

- Write number of pigs in each pen by pig type (number of sows, number of piglets, number of grower pigs - intent for sows/adults and for grower pigs)

- Mark location of water point/s and type of water point/s
