# Supplementary material for: Assessing the welfare of smallholder pigs in Vietnam using a pilot protocol
Source: Trop Anim Health Prod. 2025 Sep 23;57(8):392. doi: 10.1007/s11250-025-04602-5 (PMC12457531; doi:10.1007/s11250-025-04602-5)
Supplement: Supplementary file 2 — Supplementary Material 2 [file 11250_2025_4602_MOESM2_ESM.docx]

**SUPPLEMENTARY INFORMATION 2**

**Table**. Sources of advice and capital and knowledge of animal welfare reported by 110 smallholder pig herds in two districts of Hao Binh province, Vietnam in 2022

| **Variable** | **Da Bac (n=55)** | **Lac Son (n=55)** | **Overall (n=110)** |
| --- | --- | --- | --- |
| **Source of information/advice on how to improve the feeding of pigs*** | | | |
| Family and close friends | 39 (70.9) | 41 (74.5) | 80 (72.7) |
| Neighbours | 22^a^ (40.0) | 44^b^ (80.0) | 66 (60.0) |
| Community leader | 0 (0.0) | 0 (0.0) | 0 (0.0) |
| Government officer/veterinarian | 3 (5.5) | 2 (3.6) | 5 (4.5) |
| Radio or television | 1^a^ (1.8) | 9^b^ (16.4) | 10 (9.1) |
| Internet | 1^a^ (1.8) | 12^b^ (21.8) | 13 (11.8) |
| Trainings on pig raising | 12 (21.8) | 12 (21.8) | 24 (21.8) |
| Other | 1 (1.8) | 7 (12.7) | 8 (7.3) |
| **Source of money to build/repair pens*** |  |  |  |
| Use saved money | 46 (83.6) | 52 (94.5) | 98 (89.1) |
| Borrow from family or friend | 5 (9.1) | 5 (9.1) | 10 (9.1) |
| Borrow from bank | 10 (18.2) | 4 (7.3) | 14 (12.7) |
| Access from farmer cooperative | 0 (0.0) | 0 (0.0) | 0 (0.0) |
| Borrow from Women Union | 2 (3.6) | 0 (0.0) | 2 (1.8) |
| **Source of money to buy feed for pigs*** |  |  |  |
| Use saved money | 53^a^ (96.4) | 42^b^ (76.4) | 95 (86.4) |
| Borrow from family or friend | 9 (16.4) | 6 (10.9) | 15 (13.6) |
| Borrow from bank | 4 (7.3) | 3 (5.5) | 7 (6.4) |
| Access from farmer cooperative | 0 (0.0) | 0 (0.0) | 0 (0.0) |
| Buy on credit from feed suppliers | 4^a^ (7.3) | 25^a^ (45.5) | 29 (26.4) |
| **Have you ever heard of the term animal welfare?** | |  |  |
| No | 54 (98.2) | 54 (98.2) | 108 (98.2) |
| Yes | 1 (1.8) | 1 (1.8) | 2 (1.8) |
| **Do you want to know about animal welfare?** |  |  |  |
| No | 7 (12.7) | 15 (27.3) | 22 (20.0) |
| Yes | 48 (87.3) | 40 (72.7) | 88 (80.0) |
| * Multiple choice question  ^a, b^ Different characters in the same row means statistically different (P<0.05) | | | |
